# A rare case of syphilitic hepatic inflammatory pseudotumor misdiagnosed as liver metastasis: Case report and mini-review

**DOI:** 10.1097/MD.0000000000049413

**Published:** 2026-06-19

**Authors:** Jing Wang, Yan Wang, Ye Zhao, Shuping Li

**Affiliations:** a Department of Gansu Provincial Hospital Radiotherapy Centre-Radiotherapy Department Ward 2, Lanzhou, China; b Department of General Surgery, The Third People’s Hospital of Gansu Province, Lanzhou, China.

**Keywords:** diagnosis and treatment, liver metastases, syphilitic hepatic inflammatory pseudotumor

## Abstract

**Rationale:**

Syphilitic hepatic inflammatory pseudotumor is an exceedingly rare benign hepatic manifestation of syphilis. Due to its clinical and radiological resemblance to malignant liver tumors, it frequently poses a diagnostic challenge, often leading to potential misdiagnosis as metastatic disease.

**Patient concerns:**

A 48-year-old male truck driver presented with a 1-month history of fatigue, decreased appetite, and persistent right upper abdominal discomfort, accompanied by a 5-kg weight loss.

**Diagnoses:**

Physical examination revealed tenderness in the right upper abdomen. Laboratory tests showed elevated liver enzymes and systemic inflammatory markers. Imaging (US, CEUS, and computed tomography) initially suggested metastatic liver tumors. However, positive syphilis serology (RPR 1: 780; TPPA positive) and ultrasound-guided liver biopsy, which showed lymphoplasmacytic infiltration and interlobular fibrosis, confirmed the diagnosis of syphilitic hepatic inflammatory pseudotumor.

**Interventions:**

The patient received a 3-week course of intramuscular penicillin G (2.4 million units weekly).

**Outcomes:**

Following treatment, abdominal symptoms resolved, liver function normalized, and the rapid plasma reagin titer decreased to 1: 100.

**Lessons:**

This case emphasizes the “great imitator” nature of syphilis in hepatic lesions. Clinicians should include syphilis in the differential diagnosis of atypical hepatic masses, even in patients without high-risk histories, to avoid unnecessary invasive procedures or misdiagnosis.

## 1. Introduction

Syphilitic hepatic inflammatory pseudotumor is a rare benign lesion caused by Treponema pallidum infection, which can manifest at any stage of syphilis.^[1]^ Although inflammatory pseudotumors are characterized by tumor-like masses composed of inflammatory cells and myofibroblasts, their association with syphilis is infrequently documented. The clinical presentations are often nonspecific, mimicking primary or metastatic malignancies, which underscores the importance of early diagnostic awareness and appropriate serological screening to prevent clinical delays.^[2]^

## 2. Ethical approval

Ethical approval was waived by the Ethics Committee of Gansu Provincial Hospital because this study is a retrospective case report and does not involve any experimental intervention. Written informed consent was obtained from the patient for publication of clinical details and images.

## 3. Case summary

A 48-year-old male was admitted due to a 1-month history of right upper abdominal pain, fatigue, and significant weight loss (5 kg). The patient denied a history of infectious diseases, including hepatitis and syphilis, as well as high-risk sexual behaviors. Physical examination was unremarkable except for right upper quadrant tenderness.

Laboratory investigations revealed significantly elevated liver-associated enzymes, including alkaline phosphatase (ALP) 845 U/L (reference range, 50–135 U/L), alanine aminotransferase (ALT) 327 U/L (reference range, 7–40 U/L), and aspartate aminotransferase (AST) 154 U/L (reference range, 13–35 U/L). Systemic inflammation was indicated by an elevated C-reactive protein (CRP) level of 67.2 mg/L (reference range, < 5 mg/L) and an increased erythrocyte sedimentation rate of 48 mm/h (reference range, 0–20 mm/h for males). Serological screening for human immunodeficiency virus and viral hepatitis was negative. However, syphilis-specific tests were reactive, with a treponemal antibody signal-to-cutoff ratio (S/CO) of 111 (reference range, nonreactive < 1.0) and a rapid plasma reagin (RPR) titer of 1: 780 (reference range, nonreactive). Neurosyphilis was excluded via negative cerebrospinal fluid testing.

**Figure 1.**
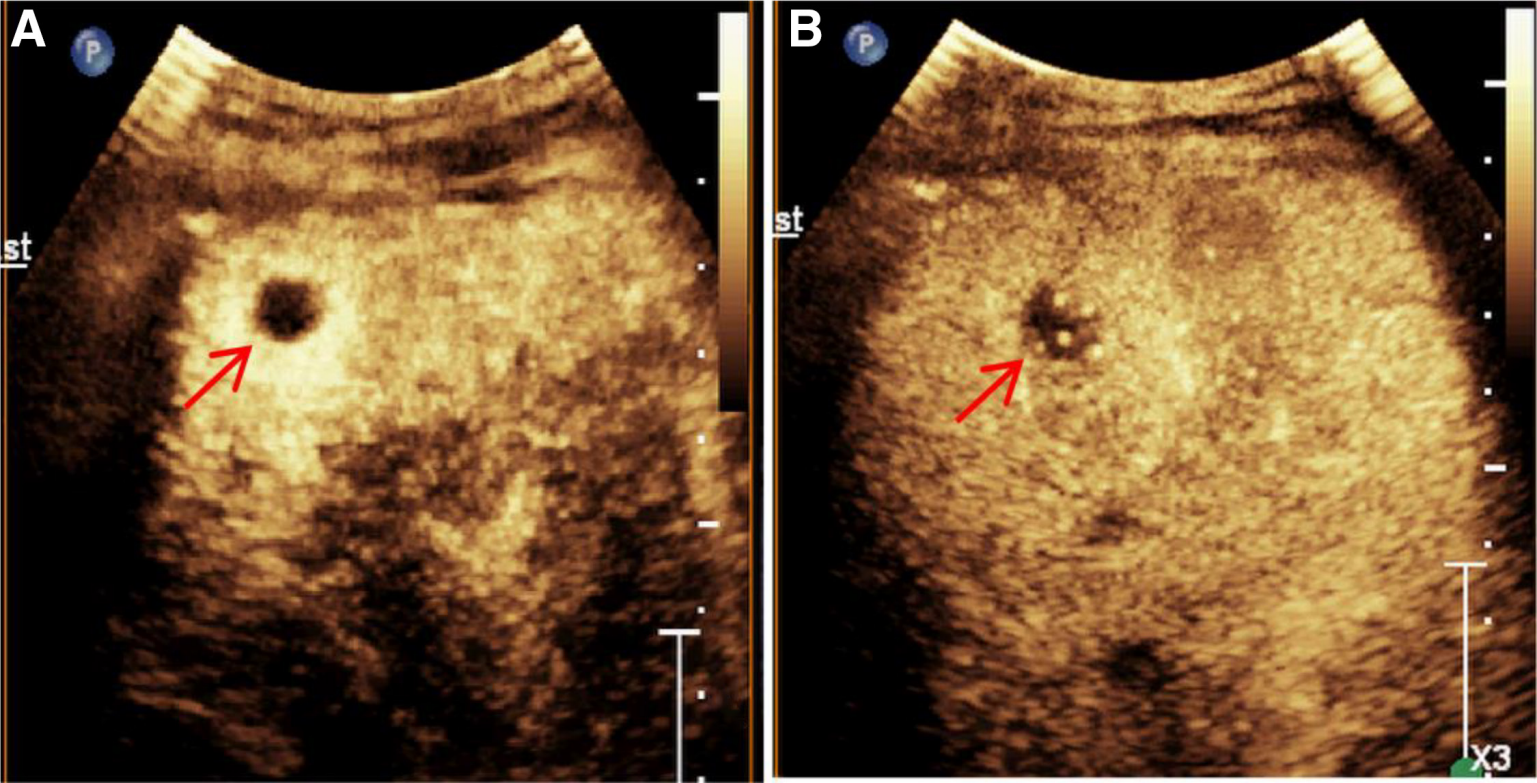
CEUS images: (A) At 19 s after intravenous injection of the contrast agent SonoVue, the lesion reached peak enhancement, displaying heterogeneous thick-ring hyperenhancement around the periphery. (B) In the delayed phase, the lesion showed continuous washout of the contrast agent, resulting in heterogeneous hypoenhancement. The red arrows indicate the lesion sites. CEUS = contrast-enhanced ultrasound, CT = computed tomography.

**Figure 2.**
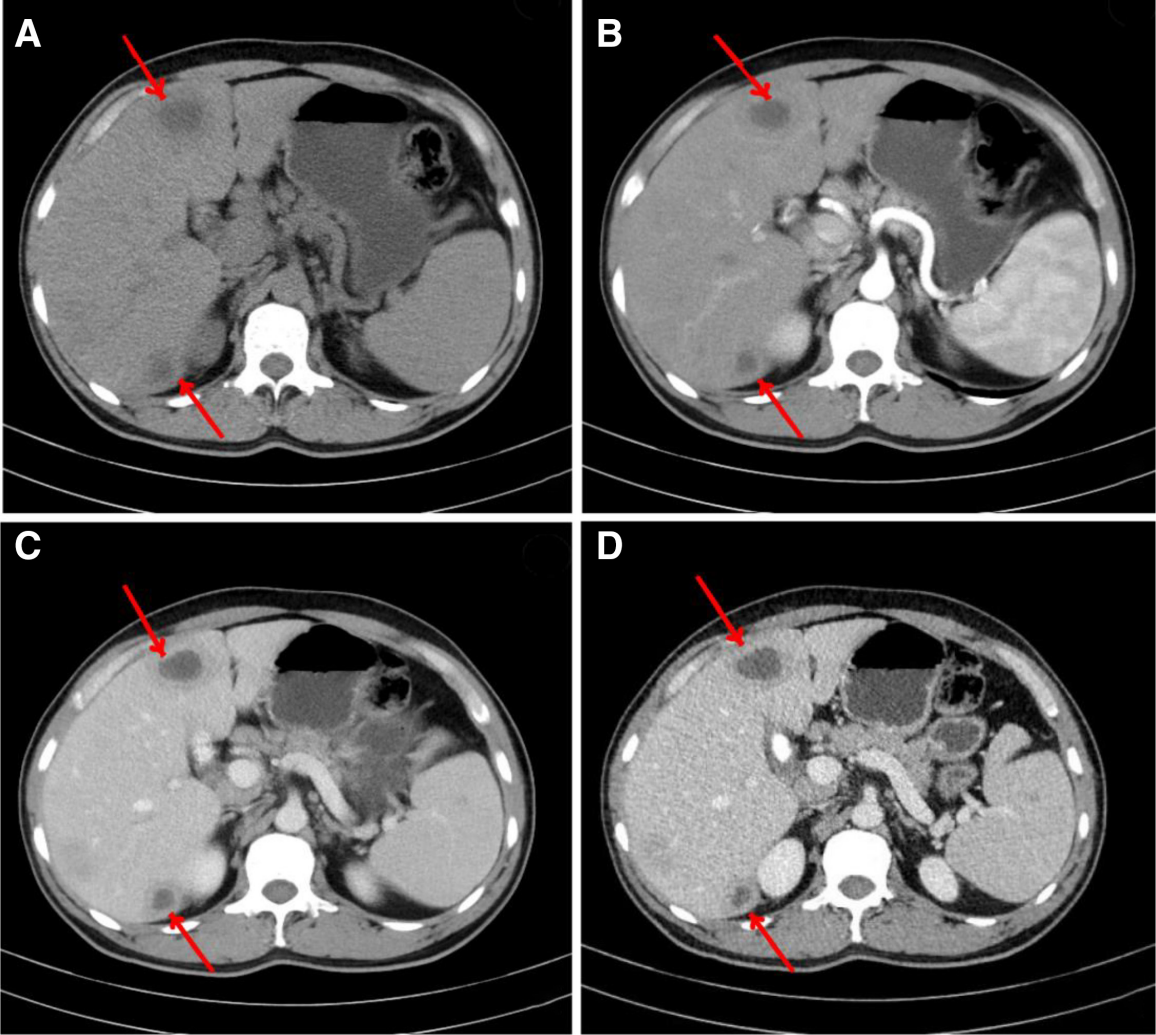
Contrast-enhanced abdominal CT: (A) Plain CT scan revealed multiple ring-shaped lesions with densities lower than that of the liver parenchyma and clear margins. (B) During the arterial phase, the lesions exhibited ring-like hypoenhancement. (C and D) In the equilibrium phase, contrast agent washout was observed. Red arrows indicate the location of the lesions. CT = computed tomography.

Imaging studies, including contrast-enhanced ultrasound (CEUS) and abdominal computed tomography (CT), revealed multiple hypodense/hypoechoic lesions with “thick-ring” enhancement and subsequent washout, characteristics typically suggestive of metastatic hepatocellular carcinoma (Figs. 1 and 2). Chest CT revealed no pulmonary nodules, and both upper and lower gastrointestinal endoscopies identified no primary tumor.

**Figure 3.**
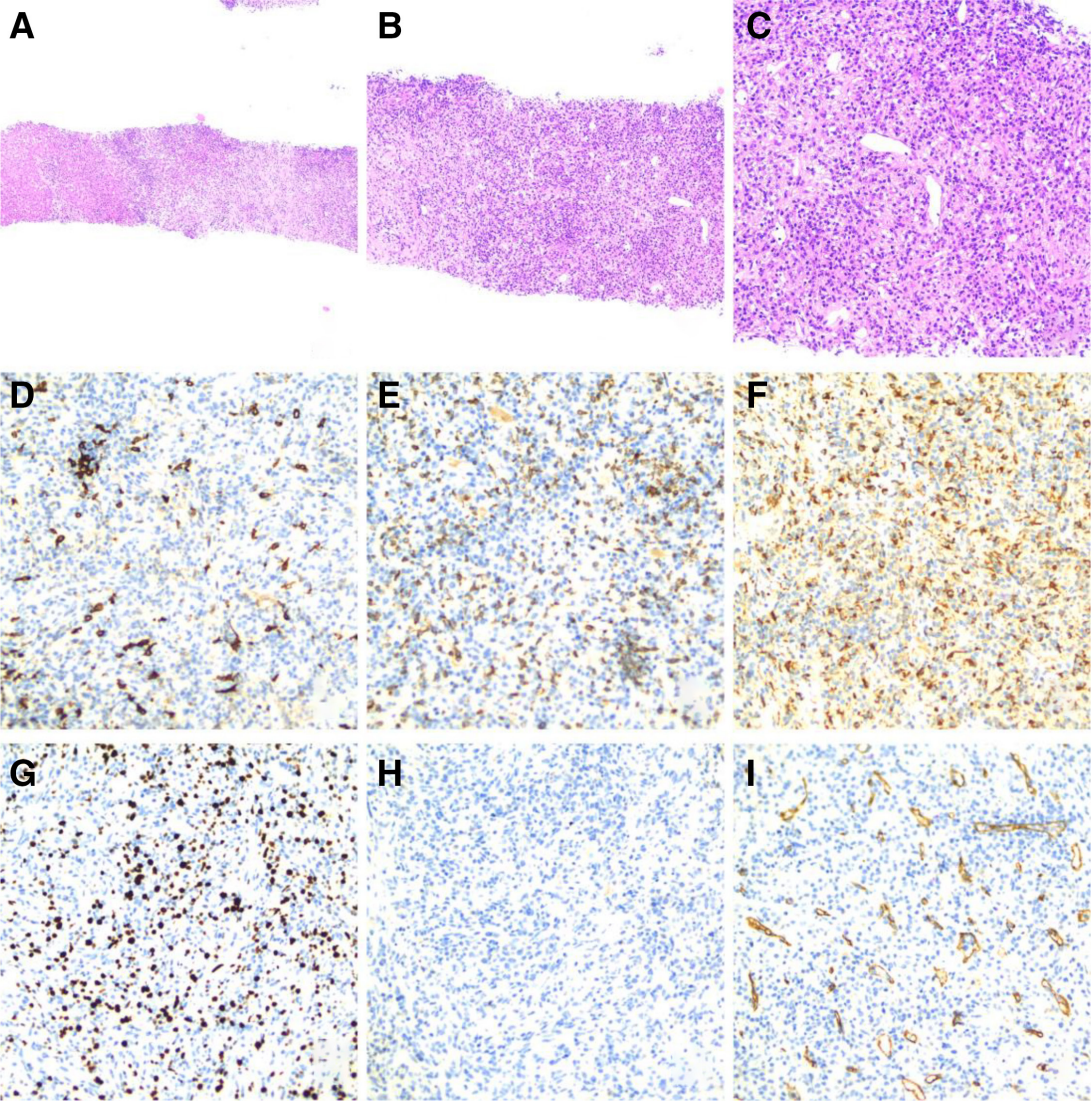
Pathological findings of fine-needle aspiration for focal hepatic lesion. (A) Prominent interlobular fibrosis with dense infiltration of lymphocytes and histiocytes (H&E stain, magnification power: ×100). (B) H&E stain, magnification power: ×200. (C) H&E stain, magnification power: ×400. Immunohistochemical staining showed: (D) CD20 (+) (B lymphocytes). (E) CD3 (+) (T lymphocytes). (F) CD68 (+). (G)Ki-67 labeling is approximately 30%. (H)Glypican-3 (−). (I) Hepatic stromal sinuses show CD34 (+).

Definitive diagnosis was achieved through ultrasound-guided liver biopsy. Histopathologic examination revealed prominent interlobular fibrosis and a dense plasma cell-rich inflammatory infiltrate composed predominantly of lymphocytes, plasma cells, and histiocytes. No definite obliterative endarteritis was identified in the sampled biopsy tissue. Immunohistochemical studies showed positivity for CD20, CD3, and CD68, supporting a mixed reactive inflammatory infiltrate. Ki-67 labeling was observed in approximately 30% of cells, and CD34 highlighted stromal-sinusoidal vascular structures. However, the lesion was negative for SMA, CD21, and CD23, which argued against inflammatory myofibroblastic tumor. Furthermore, negative results for markers like CD21, CD23, and Glypican-3 are essential to exclude inflammatory pseudotumor-like follicular dendritic cell sarcoma and primary hepatic malignancies. Although silver staining, immunohistochemistry for Treponema pallidum, or PCR were not performed on the biopsy tissue, the overall morphologic, immunophenotypic, serologic, and therapeutic findings supported the diagnosis of syphilitic hepatic inflammatory pseudotumor. Figure 3 shows images of hematoxylin-eosin staining at 100x, 200x, and 400x magnifications, along with images of immunohistochemical markers significant for diagnosis.

The patient received a 3-week course of intramuscular penicillin G (2.4 million units weekly). Within 2 months, the patient’s symptoms resolved completely, liver function returned to baseline, and the RPR titer decreased to 1: 100.

## 4. Discussion

### 4.1. Pathogenesis

Syphilis is a systemic infectious disease caused by Treponema pallidum, notoriously known as “the great imitator” due to its ability to manifest in diverse clinical forms across multiple organ systems.^[3,4]^ While hepatic involvement typically presents as syphilitic hepatitis characterized by diffuse inflammation, the formation of a syphilitic hepatic inflammatory pseudotumor is an exceedingly rare and localized manifestation.^[5–8]^ The term “inflammatory pseudotumor” (IPT) describes a benign, nonneoplastic mass characterized by a mixture of inflammatory cells and spindle-shaped myofibroblasts.^[9]^ While the exact pathogenesis of IPT remains partially elusive, it is widely considered a reactive process secondary to infection, trauma, or immunological triggers.^[1]^ In the setting of syphilis, the lesion may reflect a plasma cell-rich immune response, granulomatous inflammation, and fibrosis rather than true neoplasia. Although IPTs are documented in various organs, their occurrence in the liver – specifically triggered by syphilis – is rarely reported in the literature. Syphilitic hepatic inflammatory pseudotumor can manifest at any stage of syphilitic infection, regardless of the patient’s immune status.^[10]^

### 4.2. Literature review methodology

A nonsystematic PubMed search was performed using the terms “syphilitic hepatic inflammatory pseudotumor,” “luetic hepatitis,” and “inflammatory pseudotumor liver syphilis” to identify relevant case reports for contextualization. Reference lists of the retrieved articles were manually screened to identify additional reports of syphilitic hepatic inflammatory masses or pseudotumor-like lesions. Published reports remain scarce. The available literature mainly consists of isolated case reports and 1 small case series, underscoring the rarity of this entity and the practical value of concise case-based synthesis. Table 1 summarizes the principal clinical, radiologic, serologic, therapeutic, and outcome data from the published cases cited in this discussion.^[5,11–13]^

**Table 1:** Clinical characteristics and outcomes of syphilitic hepatic inflammatory pseudotumor cases.

Source/Author (year)	Patient Characteristics (age/sex/HIV)	Clinical presentation	Key imaging characteristics	Syphilis Serology	Histopathology (key features/IHC; TP confirmation)	Treatment Regimen	Outcome
Planella-Fontanillas (2024)^[5]^	59 / M / HIV-negative	Headache, jaundice, weight loss (25kg), palmoplantar 39macular lesions.	CT: 3 hepatic nodular lesions, enlarged intraabdominal lymph nodes (mimicking metastases); MRI: Altered signal in frontal bone.	RPR 1:1024, TPPA (+)	Plasma cell-rich inflammation with noncaseating granulomas and fibrosis; TP confirmation: NR	Benzathine penicillin G (2.4 MU/week for 3 wk).	Complete resolution of lesions; clinical cure.
Hagen et al Case 1 (2014)^[11]^	51 / M / HIV-positive	Asymptomatic; incidental finding of hepatic mass.	Two solid hepatic lesions, clinically suspicious for malignancy.	RPR 1:1028	Spindle cell proliferation, neutrophils, and abscess formation; IHC: T. pallidum (+).	Penicillin G.	Significant reduction/disappearance of lesions on follow-up.
Hagen et al Case 2 (2014)^[11]^	53 / M / HIV-positive	Constitutional symptoms, hepatosplenomegaly.	Multiple lesions in both liver and spleen.	RPR (+) (titer NR)	Storiform spindle cell infiltration with microabscesses. TP confirmation: NR	Penicillin G (3 doses).	Normalization of imaging posttreatment.
Hagen et al Case 3 (2014)^[11]^	47 / M / HIV-positive	Discovery of multiple mass lesions.	>50 ring-enhancing hepatic lesions; pulmonary nodules.	RPR 1:64	Granulomas and ericholangitis/cholangitis. TP confirmation: NR	Penicillin G (3 doses).	Complete resolution of all lesions.
DeRoche & Huber (2017)^[12]^	64 / M / HIV-negative	Anorexia, malaise, diarrhea, palmoplantar rash.	PET-CT: Multiple hypermetabolic hepatic lesions (high SUV), mimicking metastatic disease.	RPR (+) (titer NR)	Dense lymphoplasmacytic (plasma cell-rich) infiltrate; Numerous spirochetes reported (method NR)	Penicillin.	Improvement in clinical symptoms and imaging findings.
Mahto et al. (2006)^[13]^	44 / M / HIV-positive	Fever, abdominal pain, weight loss, palmoplantar rash.	US/CT: Hypoechoic/hypodense masses with ill-defined borders.	RPR 1:128	Fibroblast proliferation with prominent plasma cells (IPT-like); TP confirmation: NR	Doxycycline (200mg bid for 1 month).	Complete resolution of the pseudo-tumor.

HIV = human immunodeficiency virus, MU = million units, NR = not reported, TP = treponemal, TPPA = Treponema pallidum particle agglutination.

### 4.3. Clinical presentation

The clinical presentation is nonspecific. Patients may report right upper quadrant pain, fatigue, anorexia, fever, or weight loss, and biochemical testing often shows a cholestatic or mixed liver injury pattern with elevated ALP and inflammatory markers like CRP and erythrocyte sedimentation rate. Importantly, the absence of an obvious high-risk sexual history does not exclude syphilis, as illustrated by both the current patient and prior reports.^[5,12]^

### 4.4. Radiological characteristics and diagnostic pitfalls

Radiologically, syphilitic hepatic inflammatory pseudotumor often masquerades as malignancy. On US and CEUS, lesions typically appear hypoechoic with peripheral hyperenhancement and “washout” in the venous phase. Abdominal CT frequently reveals ring-like hypodense lesions that suggest metastatic disease. This “thick-ring” enhancement pattern is a known pitfall that can lead to the misdiagnosis of metastatic hepatocellular carcinoma or liver abscesses.^[14]^ Literature suggests that while MRI (T1-low, T2-high signals) provides more detail, considering the MRI SI pattern both in the literature, on T2W images, the dominant patterns are homo/inhomogeneous signal hyperintensity (60% vs 56%, respectively) and targetoid appearance with a hyperintense core (31% vs 44%). While MRI typically demonstrates T1 hypointense and T2 hyperintense lesions and may provide additional soft-tissue characterization, no imaging modality offers pathognomonic features for syphilitic hepatic inflammatory pseudotumor.^[15,16]^ Accordingly, imaging findings should always be interpreted together with serologic and histopathologic data rather than in isolation.

### 4.5. Histopathological diagnosis

Histopathologic evaluation remains central to the diagnosis of syphilitic hepatic inflammatory pseudotumor. The most informative morphologic features include a lymphoplasmacytic, often plasma cell-rich inflammatory infiltrate and variable fibrosclerotic stromal change.^[17]^ In some syphilitic lesions, obliterative endarteritis may also be present, although this feature is not uniformly identified and may be absent in limited biopsy samples. In our case, the biopsy demonstrated prominent interlobular fibrosis and a dense plasma cell-rich infiltrate composed of lymphocytes, plasma cells, and histiocytes, whereas definite obliterative endarteritis was not observed in the sampled tissue. Immunohistochemistry was useful primarily in excluding important histologic mimics. Positivity for CD20, CD3, and CD68 supported a mixed reactive inflammatory background. The lesion was negative for SMA, CD21, and CD23, which argued against inflammatory myofibroblastic tumor. Furthermore, negative results for markers like CD21, CD23, and Glypican-3 are essential to exclude inflammatory pseudotumor-like follicular dendritic cell sarcoma and primary hepatic malignancies.^[18]^ Ki-67 labeling was approximately 30%; however, in a lesion dominated by inflammatory cells, this proliferative index should be interpreted cautiously and not overread as evidence of malignancy. CD34 positivity was confined to stromal-sinusoidal vascular structures and was considered a nonspecific finding rather than evidence of a specific neoplastic phenotype. Although the strongly positive treponemal and non-treponemal serologic tests, compatible histopathologic findings, and favorable response to penicillin strongly supported the diagnosis of syphilis, direct confirmation of Treponema pallidum in tissue by silver staining, immunohistochemistry, or PCR would have further strengthened the diagnosis. These confirmatory studies were not feasible in the present case because of limited tissue availability and local technical constraints, and this should be acknowledged as a limitation of the report. Therefore, the final diagnosis was established on the basis of integrated clinicopathologic correlation, including serology, histopathology, exclusion of key mimics, and treatment response. In the absence of direct tissue confirmation of Treponema pallidum, the diagnosis in this case relied on integrated clinicopathologic correlation, including high-titer serologic reactivity, plasma cell-rich inflammatory histology, exclusion of major histologic mimics, and a marked response to penicillin therapy.^[1,5,11]^ Several hepatic lesions that may mimic syphilitic hepatic inflammatory pseudotumor have been reported in the literature.^[19–21]^ Their distinguishing clinical and pathological characteristics are summarized in Table 2.

**Table 2:** Differential diagnosis of syphilitic hepatic inflammatory pseudotumor vs hepatic metastases and other mimics.

Feature	Syphilitic hepatic inflammatory pseudotumor^[5,11–13]^	Hepatic Metastases^[19]^	Liver Abscess^[20]^	IMT^[21]^
Clinical Context	Positive syphilis serology (RPR/TPPA); often asymptomatic or mild pain.	Known primary tumor; significant weight loss; cachexia.	Fever/chills; leukocytosis; history of biliary/portal infection.	Nonspecific; occasionally associated with ALK gene rearrangement.
Tumor Markers	Usually normal AFP, CEA, CA19-9.	Often elevated (e.g., CEA, CA19-9).	Normal.	Normal.
CEUS/CT Pattern	“Thick-ring” enhancement with slow washout; often multifocal.	“Target” or “Bull’s eye” sign; rapid washout in venous phase.	“Cluster of grapes” sign; no enhancement in the necrotic center.	Variable; often shows persistent enhancement due to fibrosis.
Histopathology	Lymphoplasmacytic infiltration; interlobular fibrosis.	Malignant cells consistent with the primary source.	Neutrophilic infiltration; necrosis; pus formation.	Spindle-shaped myofibroblasts; ALK expression (50%).
IHC Markers	CD3(+), CD20(+), CD68(+); CD21(−), CD23(−).Ki-67 variable/nonspecific; TP IHC/PCR may be positive	Cytokeratins specific to primary (e.g., CK7, CK20).	N/A.	SMA (+), Vimentin (+), ALK (+/−) (variable)
Tissue confirmation	Silver stain/TP IHC/PCR may demonstrate spirochetes (often negative in small biopsies); absence should be acknowledged as a limitation	Not applicable	Not applicable	Not applicable
Response to Treatment	Rapid regression with Penicillin.	Response to chemo/radiotherapy or progression.	Response to antibiotics and drainage.	Variable; may require surgical resection or steroids.

CEUS = contrast-enhanced ultrasound, CT = computed tomography, IMT = Inflammatory myofibroblastic tumor, RPR = rapid plasma reagin, TPPA = treponema pallidum particle agglutination.

### 4.6. Treatment and outcome

Unlike malignant tumors requiring surgery or chemotherapy, syphilitic hepatic inflammatory pseudotumor responds remarkably well to antibiotic therapy. Penicillin G remains the first-line treatment, leading to rapid symptomatic relief and normalization of liver enzymes.^[22]^ In our case, a 3-week course of penicillin resulted in a significant decline in RPR titers and complete resolution of symptoms. Prior reports similarly support radiologic or clinical regression after antimicrobial therapy, reinforcing the value of “diagnostic treatment” – if syphilis is suspected, a trial of antibiotics can serve as both a therapeutic measure and a diagnostic confirmation, potentially sparing the patient from invasive surgery.^[5,11,12]^

## 5. Conclusion

In conclusion, clinicians must maintain a high index of suspicion for syphilis when encountering atypical hepatic masses, especially when systemic workup fails to identify a primary malignancy, even without traditional risk factors. The integration of serological screening into the routine workup of “metastatic-like” liver lesions can facilitate early diagnosis and curative treatment. Future cases should follow structured protocols with serial imaging to better document the radiological regression of these lesions posttreatment.

## Author contributions

**Funding acquisition:** Jing Wang.

**Investigation:** Yan Wang, Ye Zhao.

**Methodology:** Ye Zhao.

**Writing** – **original draft:** Jing Wang.

**Writing** – **review & editing:** Shuping Li.
